# Effect of continuous intraoperative intravenous dexmedetomidine infusion on postoperative delirium in elderly patients undergoing lower extremity orthopedic surgery under neuraxial anesthesia: a prospective single-center controlled clinical trial

**DOI:** 10.1186/s12871-026-03609-1

**Published:** 2026-01-20

**Authors:** Shangjun Gong, Peng Xia, Zhixiu Ye, Keyi Wu, Tianming Ma, Jun Yan, Yiqiao Wang

**Affiliations:** 1 Anhui No.2 Provincial People’s Hospital Clinical College of Anhui Medical University, Hefei, Anhui China; 2 Lujiang County People’s Hospital, Lujiang, Anhui China; 3 Anhui No.2 Provincial People’s Hospital, Hefei, Anhui China; 4 The Fifth Clinical Medical College of Anhui Medical University, Hefei, Anhui China

**Keywords:** Postoperative delirium, Dexmedetomidine, Lower extremity orthopedic surgery, Neuraxial anesthesia, Elderly patients

## Abstract

**Background:**

Postoperative delirium (POD) is a common and serious complication in elderly patients undergoing lower extremity orthopedic surgery, associated with increased morbidity and mortality. This study aims to evaluate the effect of intraoperative dexmedetomidine on POD incidence and severity in elderly orthopedic patients.

**Methods:**

This prospective, single-center, randomized controlled trial enrolled patients aged ≥ 65 years scheduled for lower extremity orthopedic surgery under neuraxial anesthesia. Patients were randomly assigned to receive either dexmedetomidine (Group D) or placebo (Group C) in a 1:1 ratio. The primary outcome was the incidence of POD within 7 days postoperatively, assessed using the Confusion Assessment Method (CAM). Secondary outcomes included the severity of delirium, length of hospital stay, incidence of postoperative nausea and vomiting (PONV), postoperative visual analog scale (VAS) pain scores at 6 h, 12 h, 24 h, and 48 h, patient-controlled analgesia (PCA) usage, sufentanil consumption, and other adverse events.

**Results:**

A total of 200 patients were randomized, with 92 in Group D and 91 in Group C completing the study. The incidence of POD was significantly lower in Group D (9.78%) compared to Group C (20.88%) (χ² = 4.35, *p* = 0.037). The severity of delirium was also lower in Group D (Z = -2.07, *p* = 0.038). There were no significant differences between groups in terms of PONV incidence (*p* = 0.641), VAS scores at any time point (all *p* > 0.05), PCA usage (*p* = 0.532), sufentanil consumption (*p* = 0.591), or other adverse events (*p* > 0.05).

**Conclusion:**

Intraoperative dexmedetomidine infusion significantly reduces the incidence and severity of POD in elderly patients undergoing lower extremity orthopedic surgery. This intervention appears safe and effective, with no significant increase in adverse events or impact on postoperative pain management.

**Supplementary Information:**

The online version contains supplementary material available at 10.1186/s12871-026-03609-1.

## Introduction

Postoperative delirium (POD) is a common and serious complication in elderly patients, particularly following major surgeries such as lower limb orthopedic procedures. Characterized by acute fluctuations in attention, cognition, and consciousness, POD is associated with prolonged hospital stays, increased healthcare costs, and higher morbidity and mortality rates [1, 2]. The aging population, with its heightened vulnerability to neuroinflammation and neurotransmitter dysregulation, underscores the need for effective preventive strategies [3].

Dexmedetomidine, a highly selective α2-adrenergic agonist, has emerged as a promising agent for reducing the incidence of POD [4]. Its sedative, analgesic, and anxiolytic properties, coupled with its minimal impact on respiratory function, make it an attractive option for intraoperative use [5]. Recent studies suggest that continuous intraoperative infusion of dexmedetomidine may mitigate neuroinflammatory responses and stabilize hemodynamics, thereby reducing the risk of delirium in elderly patients [6, 7]. Furthermore, dexmedetomidine has been shown to modulate the cholinergic system and reduce the release of pro-inflammatory cytokines, which are implicated in the pathogenesis of delirium [8, 9].

Despite these promising findings, the optimal dosing regimen and its efficacy in specific surgical contexts, such as lower limb orthopedic surgery, remain areas of active investigation. Lower limb orthopedic surgeries, often associated with significant pain and immobility, may uniquely benefit from dexmedetomidine’s dual analgesic and sedative effects [10]. However, further research is needed to clarify its role in this population.

This study aims to evaluate the impact of intraoperative dexmedetomidine infusion on the incidence and severity of POD in elderly patients undergoing lower limb orthopedic surgery. By integrating recent advancements in delirium research and anesthetic management, we seek to provide evidence-based insights into its potential benefits for this vulnerable population. We hypothesize that dexmedetomidine will reduce both the incidence and severity of POD in these patients under neuraxial anesthesia.

## Materials and methods

The study protocol was approved by the Ethics Committee of Lujiang County People’s Hospital (Ethics committee approval number: LYWZLL2022-1101) and registered with the Chinese Clinical Trial Registry (ID number: ChiCTR2400089461). This double-blind, randomized, placebo-controlled clinical trial was conducted between November 2022 and November 2023 at the People’s Hospital of Lujiang County, Anhui Province, China. Written informed consent was obtained from all patients by an anesthesiologist involved in the study.

### Participants

Patients aged 65 to 90 years scheduled for lower extremity orthopedic surgery under neuraxial anesthesia were randomly assigned to two equal groups using simple randomization: Group D (dexmedetomidine) and Group C (placebo). Inclusion criteria were: age between 65 and 90 years; no contraindications to lower limb orthopedic surgery or neuraxial anesthesia; ASA physical status classification of II or III; and estimated surgery duration > 1 h. Exclusion criteria included coagulation dysfunction, cardiopulmonary dysfunction, preoperative epilepsy or dementia, and arrhythmia.

### Sample size calculation

The sample size was calculated based on the primary outcome, the incidence of POD. According to the pilot study results, we assumed a 30% incidence of POD in the control group and a 13% incidence in the intervention group. With a significance level of 0.05 and a power of 80%, the required sample size was determined to be 88 patients per group. Considering a 10% dropout rate, we aimed to recruit 100 patients per group, totaling 200 patients, to ensure adequate power for the analysis.

### Randomization

Patients were randomly assigned to either Group D or Group C using simple randomization. The randomization sequence was computer-generated, and allocation details were concealed in sequentially numbered, opaque envelopes. An independent nurse anesthetist opened the envelopes to assign interventions.

### Blinding

Study drugs (dexmedetomidine or saline) were prepared by an independent nurse anesthetist as identical colorless solutions with matching labels. Throughout the entire research process, all participants, including patients, anesthesiologists, nurses, and outcome assessors were blinded to group assignments.

###  Anesthesia management

The patient was placed in the left lateral decubitus position under standard monitoring (ECG, SpO₂, NIBP). After thorough skin disinfection and local anesthesia, an 18-gauge Tuohy needle was inserted at the L2-L3 intervertebral space using the loss-of-resistance technique. A 25-gauge Whitacre spinal needle was then introduced through the epidural needle until free flow of clear cerebrospinal fluid was observed. Hyperbaric bupivacaine 0.5% (1.2–1.5 mL) was slowly injected over 30 s to achieve a sensory block below the T10 dermatome. Following spinal anesthesia administration, a 20-gauge epidural catheter was inserted 4–5 cm cephalad into the epidural space and secured. The patient was then carefully repositioned supine.

Postoperative assessment confirmed regression of the sensory block to below T12, as determined by pinprick testing. Neurological evaluation demonstrated intact consciousness (Glasgow Coma Scale 15) and preserved motor function (Bromage score 0). The patient exhibited appropriate responses to verbal commands, including purposeful movements (nodding, hand grip). Oxygen saturation (SpO₂) measured by pulse oximetry upon leaving the PACU was maintained at or above preoperative baseline values (≥ 96%).

All patients received standardized postoperative analgesia via intravenous patient-controlled analgesia (PCA) pump programmed to deliver sufentanil citrate (2 µg/kg) in 0.9% normal saline (total volume 100 mL), with individualized bolus and basal infusion rates to maintain adequate analgesia. Supplemental analgesia with parecoxib 40mg intravenous was administered as needed. Breakthrough pain (VAS ≥ 4) was treated with rescue sufentanil (5 µg intravenous bolus), with reassessment after 15 min.

### Interventions

In Group D, patients received an intravenous infusion of dexmedetomidine hydrochloride (diluted to 4 µg/mL with 0.9% normal saline) after lumbar puncture. A peripheral venous loading dose of 1 µg/kg was administered over 10 min, followed by continuous infusion at 0.2 µg/(kg·h) until the end of surgery. In Group C, patients were infused with an equal volume of 0.9% normal saline per kilogram of body weight as a placebo. The doses of dexmedetomidine and saline were calculated based on the patients’ actual body weight.

### Outcome measures

The primary outcome was the incidence of POD within 7 days postoperatively, assessed using the Confusion Assessment Method (CAM) [11]. Secondary outcomes included delirium severity based on confusion assessment method-severity (CAM-S) short form severity scores [12], length of hospital stay, incidence of PONV, VAS pain scores at 6 h, 12 h, 24 h, and 48 h, patient-controlled analgesia (PCA) usage, sufentanil consumption, and other adverse events. CAM assessments were performed at 8:00, 12:00, 16:00, 20:00, and 22:00 on postoperative days 1–7. The CAM assesses four key features: (1) acute onset, (2) inattention, (3) disorganized thinking, and (4) altered consciousness. CAM-S scores (range: 0–7) were categorized by severity: 0 (none), 1–2 (mild), 3–5 (moderate), ≥ 6 (severe). All assessors completed standardized CAM training and passed a certification test before study initiation. Patients with severe delirium (CAM-S ≥ 3) were administered haloperidol 1–2 mg IV under the supervision of a consulting psychiatrist, with doses repeated as clinically indicated. For patients unable to self-report pain (e.g., due to delirium), the Behavioral Pain Scale (BPS) was used.

### Statistical analysis

The continuous data with normal distribution were represented by Mean ± SD, and the comparison between the two groups was performed by the T-test of two independent samples. For non-normally distributed continuous variables, data were presented as median (Q1, Q3) and analyzed using the Mann-Whitney U test, with effect sizes reported as Hodges-Lehmann median difference (HLMD) and 95% confidence intervals. The classified data are represented by n (%) and were analyzed using the Chi-square test or Fisher exact probability method when appropriate. Baseline balance was assessed using standardized mean differences (SMD), with SMD < 0.1 considered negligible. For the primary outcome, subgroup analysis was performed using ordinal logistic regression to assess the impact of different variables on the incidence of POD within 7 days after surgery. For statistical analyses, SPSS statistical software (version 16.0, SPSS Inc., Chicago, IL) was used. A *P* value < 0.05 was considered statistically significant.

## Results

### Participant flow and baseline characteristics

**Fig. 1:**
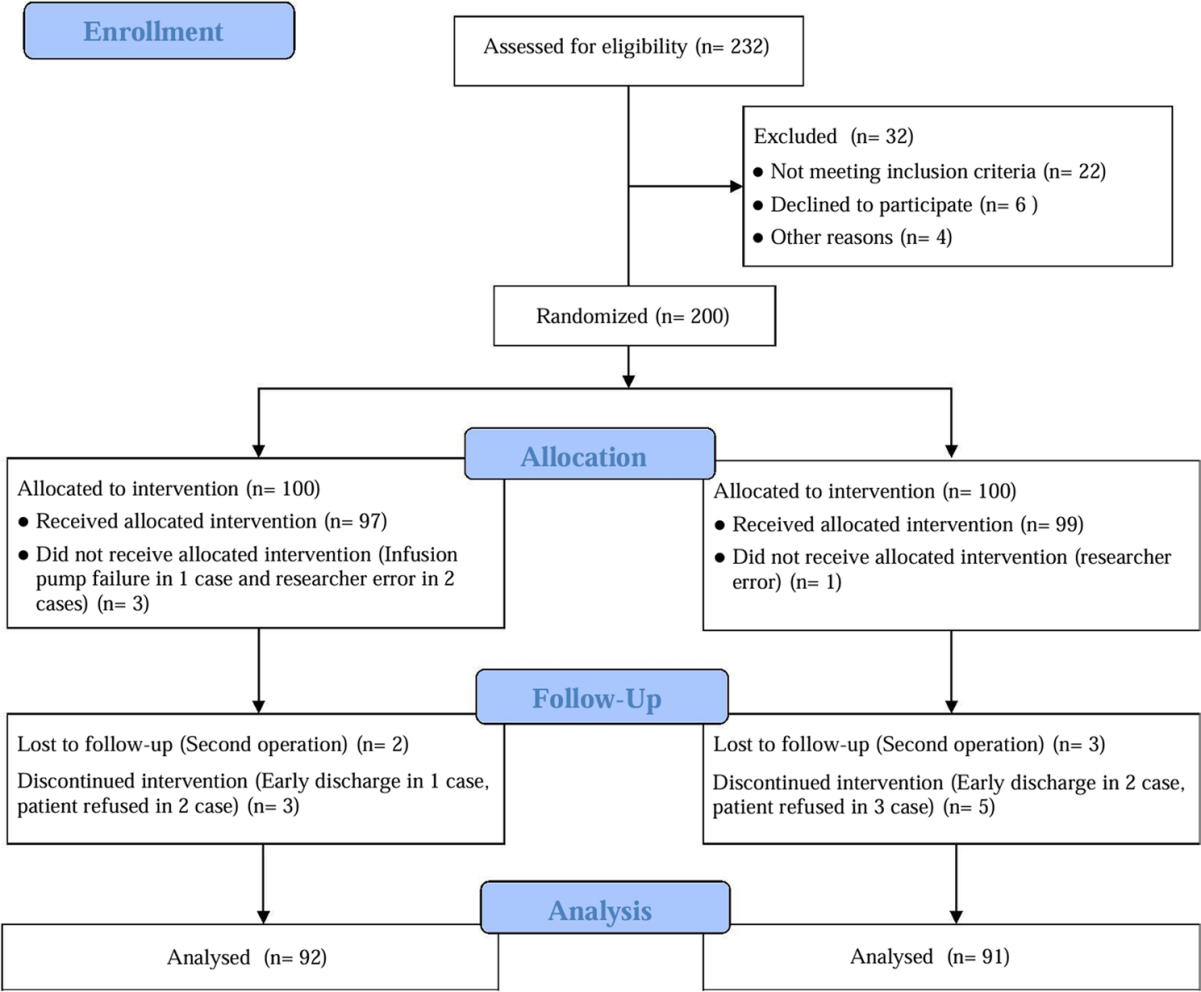
Study Flow Diagram. Caption: The diagram illustrates the flow of participants through the different stages of the study, including screening, allocation, intervention, and outcome assessment. Group D and Group C represent the two study groups with distinct interventions

A total of 232 patients were assessed for eligibility, of which 32 were excluded (22 did not meet inclusion criteria, 6 declined to participate, and 4 for other reasons). The remaining 200 patients were randomized into two groups: the dexmedetomidine group (Group D, *n* = 100) and the Placebo group (Group C, *n* = 100). During the intervention phase, 3 patients in Group D and 1 patient in Group C did not receive the allocated intervention due to infusion pump failure or researcher error. At follow-up, 2 patients in Group D and 3 patients in Group C were lost to follow-up, while 3 patients in Group D and 5 patients in Group C discontinued the intervention due to early discharge or patient refusal. Figure 1 shows the flow of participants through the study. Ultimately, 92 patients in Group D and 91 patients in Group C were included in the final analysis. Baseline demographic and clinical characteristics are summarized in Table 1. The mean age of the participants was 76.18 ± 6.25 years, with no significant differences between Group D (76.00 ± 6.20 years) and Group C (76.36 ± 6.33 years) (*SMD* = 0.057, *p* = 0.696). The majority of patients underwent femur surgery (56.28%), followed by hip surgery (18.03%). There were no significant differences in gender distribution, BMI, surgical site, anesthesia method, or operation duration between the two groups (all *SMD* < 0.2, *p* > 0.05).

**Table 1:** Demographics and clinical characteristics in the two groups of the study

Variables	Total (*n* = 183)	Study Group		SMD
		Group D (*n* = 92)	Group C (*n* = 91)	
Gender, n(%)				
Male	65 (35.52)	37 (40.22)	28 (30.77)	−0.197
Female	118 (64.48)	55 (59.78)	63 (69.23)	0.197
Age, years, mean SD	76.18 ± 6.25	76.00 ± 6.20	76.36 ± 6.33	0.057
BMI, kg•m^− 2^, mean SD	21.90 ± 2.03	21.99 ± 2.12	21.82 ± 1.95	−0.086
Surgery spot, n (%)				
Ankle	12 (6.56)	5 (5.43)	7 (7.69)	0.089
Patella	15 (8.20)	6 (6.52)	9 (9.89)	0.125
Femur	103 (56.28)	53 (57.61)	50 (54.95)	−0.053
Tibiofibula	20 (10.93)	9 (9.78)	11 (12.09)	0.075
Hip	33 (18.03)	19 (20.65)	14 (15.38)	−0.138
Anesthesia method, n (%)				
SA	65 (35.52)	32 (34.78)	33 (36.26)	0.031
CSEA	118 (64.48)	60 (65.22)	58 (63.74)	−0.031
Operation duration, min, M (Q₁, Q₃)	121.00 (89.00, 150.00)	125.50 (90.75, 151.25)	116.00 (88.50, 149.00)	−0.104

Caption: Baseline demographic and clinical characteristics of participants in Group D and Group C. Data are presented as mean ± SD, number (percentage), or median (first quartile, third quartile)

*BMI* Body Mass Index, *SA* Spinal anaesthesia, *CSEA* Combined spinal and epidural analgesia, *SD* standard deviation, *M* Median, *Q1* First Quartile, *Q3* Third quartile, *SMD* Standardized Mean Differences

### Primary outcome

#### Incidence of POD

The incidence of POD was significantly lower in Group D (9.78%) compared to Group C (20.88%) (RR = 0.47, 95% CI: 0.22–0.98; χ² = 4.35, *p* = 0.037; Table 2).

**Table 2:** Primary outcome and secondary outcome measures of the study

Outcomes	Group D (*n* = 92)	Group C (*n* = 91)	Effect Size (95% CI)	Statistic	*P*
Delirium, n (%)	9 (9.78)	19 (20.88)	RR = 0.47 (0.22–0.98)	χ²=4.35	0.037
CAM-S, M (Q₁, Q₃)	0.00 (0.00, 0.00)	0.00 (0.00, 0.00)	HLMD = 0.00 (0.00–0.00)	Z=−2.07	0.038
LOS, M (Q₁, Q₃)	9.00 (8.00, 10.00)	9.00 (8.00, 11.00)	HLMD = 0.00 (−1.00–0.00)	Z=−1.69	0.091
PONV, n (%)	11 (11.96)	13 (14.29)	RR = 0.84 (0.40–1.77)	χ²=0.22	0.641
PCA, M (Q₁, Q₃)	15.50 (9.75, 20.25)	14.00 (10.00, 20.00)	HLMD = 1.00 (−1.00–2.00)	Z=−0.62	0.532
SF, M (Q₁, Q₃)	72.00 (66.00, 94.50)	77.00 (65.50, 95.00)	HLMD=−2.00 (−6.00–3.00)	Z=−0.54	0.591
Other AEs, n (%)	15 (16.30)	10 (10.99)	RR = 1.49 (0.70–3.13)	χ²=1.10	0.295
Hypotension	5 (5.43)	2 (2.20)			
Hyperteension	1 (1.09)	2 (2.20)			
Bradycardia	8 (8.70)	4 (4.40)			
Tachycardia	1 (1.09)	2 (2.20)			

Caption: Baseline demographic and clinical characteristics of participants in Group D and Group C. Data are presented as mean ± SD, number (percentage), or median (first quartile, third quartile)

*CAM-S* Confusion Assessment Method - Severity, *LOS* Length of Hospital Stay, *PONV* Postoperative Nausea and Vomiting, *PCA* Patient-Controlled Analgesia, *SF* Sufentanil, *AE* Adverse Event, *M* Median, *Q*_1_ First Quartile, *Q*_3_ Third Quartile, *RR* Relative Risk (reference: Group D), *HLMD* Hodges-Lehmann Median Difference (Mann-Whitney U test)

#### Subgroup Analysis 

**Fig. 2:**
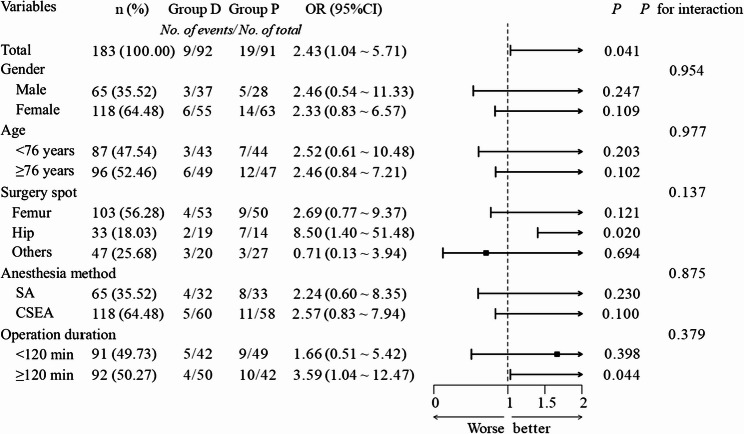
Subpopulation analysis of primary outcomes and forest plots. Caption: The forest plots display the odds ratios (OR) and 95% confidence intervals (CI) for the primary outcome (delirium) across different subgroups. SA, Spinal anaesthesia; CSEA, Combined spinal and epidural analgesia

Subgroup analyses revealed significant heterogeneity in treatment effects across various demographic and clinical subgroups (see Fig. 2). Specifically, patients undergoing hip surgery (OR = 8.50, 95% CI: 1.40–51.48; *p* = 0.020) and those with longer procedures (≥ 120 min) (OR = 3.59, 95% CI: 1.04–12.47; *p* = 0.044) had a higher incidence of delirium in Group C. However, interaction tests across all subgroups were non-significant (all *p* > 0.05).

### Secondary outcomes

#### Severity of Delirium

The severity of delirium, assessed using the CAM-S score, was significantly lower in Group D compared to Group C (*Z* = −2.07, *p* = 0.038; Table 2). The distribution of delirium cases is presented in Table 3. Ordered logistic regression analysis indicated that patients in Group D had a lower probability of experiencing higher delirium severity, with an odds ratio (OR) of 0.412 (95% CI: 0.176, 0.966; *p* = 0.041). Detailed parameter estimates are provided in Supplementary Table 1.

**Table 3:** Distribution of delirium severity based on CAM-S short form severity scores

Delirium Severity	Group D(*n* = 92)	Group C(*n* = 91)
No Delirium (0)	83 (90.2%)	72 (79.1%)
Mild (1)	4 (4.3%)	7 (7.7%)
Moderate (2)	2 (2.2%)	7 (7.7%)
Severe (≥ 3)	3 (3.3%)	5 (5.5%)

Caption: Distribution of delirium severity in Group D and Group C. The CAM-S short form severity score ranges from 0 to 7, with higher scores indicating more severe delirium.0: No delirium 1: Mild delirium, 2: Moderate delirium, ≥ 3: Severe delirium. Data are presented as frequencies (percentages)

*CAM-S* Confusion Assessment Method – Severity

The final ordinal logistic regression model significantly improved upon the intercept-only model (χ² = 4.417, *p* = 0.036), indicating that the predictors contributed significantly to the outcome. The model’s explanatory power was modest, with pseudo R-square values ranging from 0.020 to 0.034. Goodness-of-fit tests confirmed that the model fit the data well (Pearson χ² = 0.618, *p* = 0.734; Deviance χ² = 0.635, *p* = 0.728), and the parallel lines assumption was validated (χ² = 0.635, *p* = 0.728). Detailed statistical summaries are provided in Supplementary Material (Supplementary Tables 2 and Supplementary Table 3).

#### Length of Hospital Stay

The median length of hospital stay was 9 days in both Group D and Group C (HLMD = 0.00, 95% CI:−1.00–0.00; *Z* = −1.69, *p* = 0.091; Table 2).

#### PONV 

The incidence of PONV was similar between Group D (11.96%, 11/92) and Group C (14.29%, 13/91), with no significant difference observed (RR = 0.84, 95% CI:0.40–1.77; χ² = 0.22, *p* = 0.641; Table 2).

#### Postoperative Pain

**Fig. 3:**
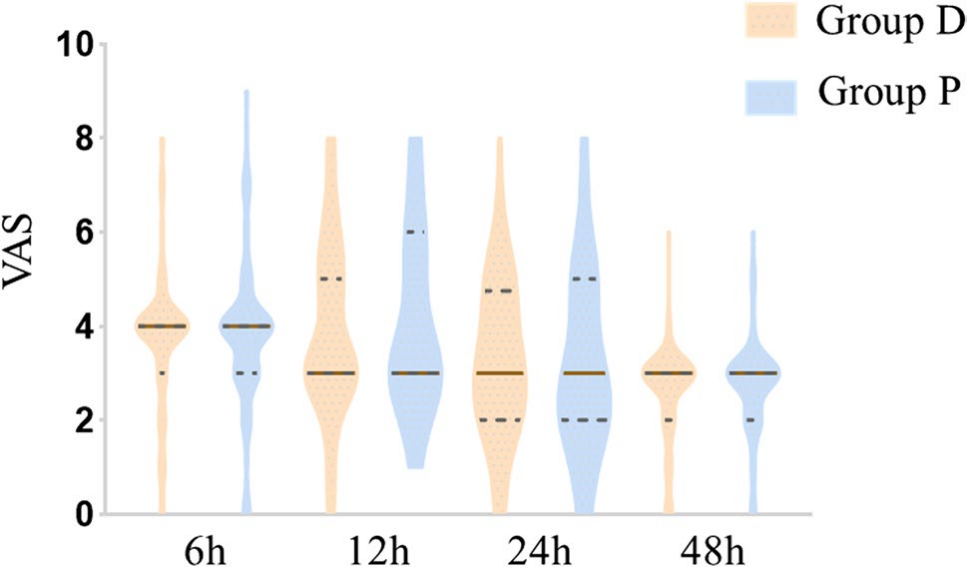
Violin plot of VAS scores at different time points after surgery. Caption: The violin plots illustrate the distribution of VAS scores at 6 h, 12 h, 24 h, and 48 h postoperatively. The median scores are indicated by horizontal lines within the plots. VAS, Visual Analogue Scale

The Visual Analogue Scale (VAS) scores were measured at 6 h, 12 h, 24 h, and 48 h postoperatively to assess pain intensity in both study groups. As illustrated in Fig. 3, the violin plots show the distribution of VAS scores at each time point. There were no significant differences in pain intensity between Group D and Group C at any time point (all *p* > 0.05). Complementing these pain assessments, PCA usage (HLMD = 1.00, 95% CI:−1.00–2.00; Z = −0.62, *p* = 0.532) and sufentanil consumption (HLMD=−2.00, 95% CI:−6.00–3.00; Z = −0.54, *p* = 0.591) also showed no significant differences (Table 2).

#### Other adverse events

The incidence of other adverse events, including hypotension, hypertension, bradycardia, and tachycardia, did not significantly differ between Group D and Group C (RR = 1.49, 95% CI: 0.70–3.13; χ² = 1.10, *p* = 0.295; Table 2). Among these, hypotension was the most common adverse event, occurring in 8.70% of Group D and 4.40% of Group C.

## Discussion

This study demonstrates that intraoperative continuous infusion of dexmedetomidine significantly reduces the incidence of POD in elderly patients undergoing lower extremity orthopedic surgery under neuraxial anesthesia. The incidence of POD in the dexmedetomidine group (9.78%) was markedly lower than in the placebo group (20.88%), supporting the potential role of dexmedetomidine as a preventive strategy for POD in this vulnerable population [1, 2].

Dexmedetomidine, a highly selective α2-adrenergic agonist, exerts its anti-delirium effects through multiple mechanisms. First, it modulates the central noradrenergic system, reducing the release of norepinephrine and stabilizing hemodynamics, which may mitigate the neuroinflammatory response associated with delirium [8, 9]. Second, dexmedetomidine enhances the activity of the cholinergic system, which is often impaired in delirium [7]. Third, its sedative and analgesic properties provide a unique advantage in managing postoperative pain and agitation, both of which are risk factors for delirium [5].

Our findings align with previous research demonstrating the efficacy of dexmedetomidine in reducing POD. For instance, a meta-analysis by Duan et al. [6] reported that perioperative dexmedetomidine significantly lowered the risk of delirium in elderly surgical patients. Similarly, Su et al. [4] found that dexmedetomidine reduced POD incidence in non-cardiac surgery patients. However, our study specifically focuses on lower extremity orthopedic surgery, a population at high risk for POD due to factors such as immobility, pain, and prolonged recovery [10, 13]. The observed reduction in POD severity, as measured by the CAM-S score, further underscores the clinical relevance of dexmedetomidine in this context.

Although the subgroup analysis did not reveal significant interactions, patients undergoing hip surgery and those with longer procedure durations (≥ 120 min) had higher delirium incidence in the placebo group, consistent with previous studies [14, 15]. Hip surgery, due to its complexity and significant impact on patient physiology, may increase the risk of POD. Additionally, prolonged surgery duration may lead to greater inflammatory responses and neurocognitive dysfunction [16]. Future research should further explore the impact of these factors on POD and identify more effective preventive strategies.

The observed safety profile of dexmedetomidine in our cohort appeared consistent with existing literature regarding its known hemodynamic effects [5]. While hypotension and bradycardia - established pharmacological effects of α2-agonists - were documented, their incidence rates showed no statistically significant elevation compared to placebo. This may suggest that the implemented dosing regimen (1 µg/kg bolus followed by 0.2 µg/kg/h maintenance) could represent a potentially tolerable option for elderly patients, though this requires verification in larger populations. Notably, the absence of between-group differences in PONV incidence and VAS pain scores implies that dexmedetomidine did not appear to aggravate these common postoperative concerns [17, 18]. However, these safety observations should be interpreted with caution given the study’s sample size limitations in detecting rare adverse events.

### Limitations

Several limitations should be acknowledged. First, this study was conducted at a single center, which may limit the generalizability of the findings. Second, the sample size, while adequate for assessing the primary outcome of delirium incidence, may limit the ability to draw definitive conclusions about safety, particularly for rare adverse events. Third, the sedative effects of dexmedetomidine could have allowed anesthesiologists to infer group assignments based on the sedation status of participants, potentially introducing bias. Future multicenter studies with larger sample sizes are needed to confirm these findings and further explore the long-term effects [19, 20]. Fourth, our postoperative pain management protocol did not incorporate multimodal analgesia, such as basal analgesics (e.g., acetaminophen or NSAIDs) or regional anesthesia techniques (e.g., adductor canal block). Given that inadequate pain control is a known risk factor for postoperative delirium, this limitation may affect the generalizability of our findings.

### Clinical implications

The results of this study have important clinical implications. Given the high morbidity and mortality associated with POD, particularly in elderly patients, the use of intraoperative dexmedetomidine represents a promising preventive strategy. Its dual benefits of reducing delirium and providing analgesia make it particularly suitable for lower extremity orthopedic surgery, where pain management is critical. However, individualized dosing and careful hemodynamic monitoring are essential to minimize potential side effects [21, 22].

## Conclusion

In conclusion, intraoperative continuous infusion of dexmedetomidine significantly reduces the incidence and severity of POD in elderly patients undergoing lower extremity orthopedic surgery under neuraxial anesthesia. These findings support the incorporation of dexmedetomidine into perioperative care protocols for high-risk populations. Further research is needed to optimize dosing regimens and evaluate long-term outcomes.

## Supplementary Information

Supplementary Material 1.

Supplementary Material 2.

## Data Availability

No datasets were generated or analysed during the current study.

## Abbreviations

CAM: Confusion Assessment Method
CAM-S: Confusion Assessment Method - Severity
VAS: Visual Analogue Scale
PONV: Postoperative Nausea and Vomiting
PCA: Patient-Controlled Analgesia
SA: Spinal Anesthesia
CSEA: Combined Spinal and Epidural Analgesia
HLMD: Hodges-Lehmann median difference
RR: Relative risk
