# Supplementary material for: Effect of continuous intraoperative intravenous dexmedetomidine infusion on postoperative delirium in elderly patients undergoing lower extremity orthopedic surgery under neuraxial anesthesia: a prospective single-center controlled clinical trial
Source: BMC Anesthesiol. 2026 Jan 20;26:134. doi: 10.1186/s12871-026-03609-1 (PMC12922267; doi:10.1186/s12871-026-03609-1)
Supplement: Supplementary file 1 — Supplementary Material 1. [file 12871_2026_3609_MOESM1_ESM.pdf]

Supplementary Material:

Supplementary Table 1: Parameter Estimates for the Ordinal Logistic Regression Model

Variable	Estimate	Standard Error	Wald χ^2	df	*p*	95% Confidence Interval
CAM = 0	Reference					
CAM = 1	1.334	0.257	26.871	1	<0.001	0.830 to 1.839
CAM = 2	1.914	0.296	41.956	1	<0.001	1.335 to 2.494
CAM = 3	2.730	0.390	49.005	1	<0.001	1.966 to 3.495
Group = 1	-0.885	0.434	4.162	1	0.041	-1.736 to -0.035
Group = 2	0[a]					

a. This parameter is set to zero as the reference category. Group D and Group P were coded as 1, 2 respectively.

Supplementary Table 2: Model Fit and Explanatory Power

Measure	Value
Model Fit	
-2 Log Likelihood (Final)	20.266
-2 Log Likelihood (Intercept Only)	24.682
Chi-Square (Model Improvement)	4.417
Degrees of Freedom	1
Significance	0.036
Pseudo R-Square	
Cox & Snell R-Square	0.024
Nagelkerke R-Square	0.034
McFadden's R-Square	0.020

Supplementary Table 3: Goodness-of-Fit Tests and Parallel Lines Assumption

Test	Chi-Square	Degrees of Freedom	Significance
Goodness-of-Fit Tests			
Pearson	0.618	2	0.734
Deviance	0.635	2	0.728
Parallel Lines Assumption			
Chi-Square	0.635	2	0.728
